# Low glucose and metformin-induced apoptosis of human ovarian cancer cells is connected to ASK1 via mitochondrial and endoplasmic reticulum stress-associated pathways

**DOI:** 10.1186/s13046-019-1090-6

**Published:** 2019-02-13

**Authors:** Liwei Ma, Jianwei Wei, Junhu Wan, Weiwei Wang, Li Wang, Yongjie Yuan, Zijun Yang, Xianzhi Liu, Liang Ming

**Affiliations:** 1 Department of Clinical Laboratory, The First Affiliated Hospital of Zhengzhou University, Zhengzhou, 450000 Henan China; 2 Department of Neurosurgery, The First Affiliated Hospital of Zhengzhou University, Zhengzhou, 450000 Henan China; 3 Department of Pathology, The First Affiliated Hospital of Zhengzhou University, Zhengzhou, 450000 Henan China; 4 Department of Interventional Neurology, The First Affiliated Hospital of Zhengzhou University, Zhengzhou, 450000 Henan China; 5 Department of Nephropathy, The First Affiliated Hospital of Zhengzhou University, Zhengzhou, 450000 Henan China; 6 Henan Medical College, Zhengzhou, 450000 Henan China

**Keywords:** Mitochondrial damage, ER stress, ASK1, Metformin, Ovarian cancer

## Abstract

**Background:**

Metformin, a first-line drug for type 2 diabetes, could induce apoptosis in cancer cells. However, the concentration of glucose affects the effect of metformin, especially low glucose in the culture medium can enhance the cytotoxicity of metformin on cancer cells. Since mitochondria and endoplasmic reticulum is vital for maintaining cell homeostasis, we speculate that low glucose and metformin-induced cell apoptosis may be associated with mitochondria and endoplasmic reticulum. ASK1, as apoptosis signaling regulating kinase 1, is associated with cell apoptosis and mitochondrial damage. This study was designed to investigate the functional significance of ASK1, mitochondria and endoplasmic reticulum and underlying mechanism in low glucose and metformin-induced cell apoptosis.

**Methods:**

An MTT assay was used to evaluate cell viability in SKOV3, OVCAR3 and HO8910 human ovarian cancer cells. Cell apoptosis was analyzed by flow cytometry. The expression of ASK1 was inhibited using a specific pharmacological inhibitor or ASK1-siRNA. Immunofluorescence was used to detect mitochondrial damage and ER stress. Nude mouse xenograft models were given metformin or/and NQDI-1, and ASK1 expression was detected using immunoblotting. In addition, subcellular fractionation of mitochondria was performed to assay the internal connection between ASK1 and mitochondria.

**Results:**

The present study found that low glucose in culture medium enhanced the anticancer effect of metformin in human ovarian cancer cells. Utilization of a specific pharmacological inhibitor or ASK1-siRNA identified a potential role for ASK1 as an apoptotic protein in the regulation of low glucose and metformin-induced cell apoptosis via ASK1-mediated mitochondrial damage through the ASK1/Noxa pathway and via ER stress through the ROS/ASK1/JNK pathway. Moreover, ASK1 inhibition weakened the antitumor activity of metformin in vivo. Thus, mitochondrial damage and ER stress play a crucial role in low glucose–enhanced metformin cytotoxicity in human ovarian cancer cells.

**Conclusions:**

These data suggested that low glucose and metformin induce cell apoptosis via ASK1-mediated mitochondrial damage and ER stress. These findings indicated that the effect of metformin in anticancer treatment may be related to cell culture conditions.

## Background

Ovarian cancer remains one of the most common gynecological tumors [1]. Most patients with ovarian cancer are diagnosed at an advanced stage of III or IV, which hinders effective treatment in the clinic [2]. The first-line chemotherapy for advanced ovarian cancer is cisplatin, but subsequent drug resistance minimizes the effectiveness of cisplatin and many other chemotherapy drugs [3]. Therefore, there is a critical need for novel approaches for the effective treatment of ovarian cancer.

Recent epidemiological evidence has shown that ovarian carcinogenesis is negatively correlated with obesity [4, 5]. Some groups have focused on “reprogramming of energy metabolism” as a hallmark of cancer and found that targeting cancer metabolism inhibits cancer cell growth [6]. Dr. Otto Warburg has previously reported that the underlying metabolism of malignant cancer is different from that of adjacent normal tissue [7] and that cancer cells are mainly dependent on glycolysis for glucose metabolism even in the presence of oxygen. Glycolysis provides ATP with low efficiency, but it supplies sufficient intermediates for the biosynthesis of nucleotides, NADPH, and amino acids [8]. Thus, a high rate of glucose uptake is required for the survival of cancer cells. As a result, the glucose level influences the effect of cancer treatment. High glucose promotes the proliferation of cancer cells, whereas reduced glucose enhances the cytotoxicity of therapeutic drugs, such as metformin, in several cancers, including ovarian cancer [9]. Moreover, Zhuang Y et al. found low glucose and metformin treatment in cancer cells leads to cell death by decreasing ATP production and inhibiting survival signaling pathways [9]. In general, the culture medium of cancer cells contains high glucose (25 mM), which is the optimal environment facilitating cancer cell growth. The normal level of serum glucose is approximately 4–6 mM, but the glucose level of cancer cell culture medium is decreased to 2.5 mM [9, 10]. Thus, caloric restriction and even starvation can effectively reduce the growth of cancer cells [11, 12]. As a biguanide drug, metformin is commonly considered as an effective treatment for type 2 diabetes, mainly due to its glucose-lowering effect [13]. Studies have confirmed that metformin increases the ratios of both ADP/ATP and AMP/ATP, resulting in a decreased cellular energy level through specific inhibition of mitochondrial respiratory-chain complex 1 [14–17]. In the response to metformin-induced energetic stress, the byproducts of mitochondrial respiration, reactive oxygen species (ROS), damage cellular components, such as mitochondria, leading to cell death in high concentrations [18]. ROS accumulation-induced cell death is associated with ASK1 [19]. ASK1 is a ubiquitously expressed MAP3K and can be activated by various stressors, such as oxidative stress, lipopolysaccharide and ATP [19]. ASK1 activation selectively results in sustained Jun N-terminal kinase (JNK) activation, which is associated with ER stress.

ER stress can be induced by glucose deprivation, and hypoglycemia induces the ER stress–unfolded protein response (UPR) system in retinal pericytes [20–23]. ER stress is usually activated in response to various stressors, including low glucose level, and either promotes cell survival or induces cell death in cancer cells [24–26]. When cells show altered glucose metabolism from glycolysis, ER stress is further exacerbated by glucose insufficiency [27]. Initiation of adaptive ER stress protects stressed cells from apoptosis through maintaining cell homeostasis. Grp78, which is also called immunoglobulin heavy chain binding protein (BiP), is a key factor in the ER stress process and is detected under conditions of ER stress-inducing agents [28, 29]. An elevated level of GRP78 not only indicates elimination of the harmful components of various stresses in cancer cells, but it may also reflect a change in cancer cell metabolism, such as the Warburg effect [30]. However, severe ER stress leads to cell death despite a high Grp78 expression levels. CCAAT/enhancer binding protein homologous transcription factor (CHOP), which is also called growth arrest and GADD153, plays a decisive role in this process [31]. The expression of CHOP in normal cells is difficult to detect, but severe ER stress induces CHOP transcription. The acute increase of CHOP expression leads to activation of the mitochondria-mediated apoptosis pathway [31]. However, the mechanism of ER stress induction under conditions of low glucose and metformin remains poorly understood.

The present study showed that human ovarian cancer cells were insensitive to metformin alone but that low glucose enhanced the cytotoxicity of metformin in these cells. Moreover, the mechanism of low glucose and metformin-induced cell apoptosis was investigated. Low glucose and metformin caused the accumulation of ROS, which led to mitochondrial dysfunction and activation of ASK1 and JNK. Activated ASK1 was involved in low glucose and metformin-induced mitochondrial dysfunction and ER stress. Sustaining ER stress induced ovarian cancer cells to undergo apoptosis. Thus, mitochondrial damage and ER stress together played a crucial role in low glucose–enhanced metformin cytotoxicity in human ovarian cancer cells. Moreover, ASK1 was involved in the anti-tumor effect of metformin in vivo. These data suggested that low glucose and metformin induce cell apoptosis via ASK1-mediated mitochondrial damage and ER stress. These findings suggested that changes in cellular conditions combined with metformin treatment may represent a novel treatment strategy for the clinical treatment of cancer. The present study aided the understanding of the mechanism of metformin resistance.

## Materials and methods

### Cell lines

SKOV3, OVCAR3 and HO8910 human ovarian cancer cells were purchased from American Tissue Culture Collection (ATCC; Rockville, MD). Cells were cultured in RPMI-1640 medium (GIBCO, Carlsbad, CA), supplemented with 10% fetal bovine serum (Invitrogen, Carlsbad, CA) at 37 °C with 5% CO2.

### Cell viability assays

SKOV3, OVCAR3 and HO8910 human ovarian cancer cells were seeded into 96-well microplates at a density of 1 × 10^4^ cells/well in 100 μl of complete medium and cultured overnight. Cells were then treated with varying concentrations of glucose and metformin for 24 h or 48 h. An MTT assay was used to detect cell viability by adding 20 μl of MTT (3-(4,5-dimethylthiazol-2-yl)-2,5-diphenyltetrazolium bromide; 5 mg/ml in PBS) to each well for 4–6 h. To each well, 150 μl of dimethyl sulfoxide was added (Beijing Chemical Industry Limited Company, China). The absorbance was detected at a wavelength of 570 nm using a Vmax Microplate Reader (Molecular Devices, Sunnyvale, CA).

### Immunofluorescent confocal laser microscopy

Cells were seeded into a 24-well microplate and incubated overnight, and cells were then treated with different concentrations of glucose and metformin for the indicated periods. Cells were washed 3 times with PBS, fixed with 4% paraformaldehyde for 20 min and blocked with 10% goat serum for 30 min. Cells were then incubated overnight at 4 °C with the following primary antibodies: PDI, GADD153, and cleaved caspase 3 (1:100). Cells were then incubated with FITC/Texas Red-conjugated secondary antibodies (1:200) (Santa Cruz Biotechnology, CA) for 30 min, and cell nuclei were stained with Hoechst 33342 (Sigma–Aldrich, St. Louis, MO) for 2 min. Cells were then washed 3 times with PBS and then visualized using confocal fluorescence microscopy.

### Reactive oxygen species (ROS) assays

The intracellular levels of reactive oxygen species (ROS) in cells treated with low glucose and metformin were measured using dichlorodihydrofluorescein diacetate (DCFH-DA) (Sigma) and flow cytometric analysis. As DCFH-DA is cell permeable, it interacts with intracellular ROS when it enters cells to generate fluorescent dichlorofluorescein. Cells (1 × 10^4^ cells/well) were seeded into 24-well microplates and incubated overnight. After treating cells with low glucose and metformin, cells were washed 3 times with cold phosphate-buffered saline (PBS) and then incubated with 5 μM DCFH-DA solution for 15 min at 37 °C. Cells were then washed 3 times with PBS and subsequently analyzed by flow cytometry.

### Western blot analysis

After harvesting cells treated with low glucose and metformin, cells were washed with cold PBS. Ice-cold RIPA buffer was added to cells, and cells were sonicated for 30 s on ice and lysed at 4 °C for 1 h. After centrifugation at 12,000 g for 45 min, the supernatant was collected. Protein concentration was detected by the Bio-Rad Protein Assay Kit (Bio-Rad, Hercules, CA). Proteins (25–45 μg) were separated by SDS electrophoresis and transferred onto PVDF membranes. PVDF membranes were blocked with nonfat dry milk in buffer for 60 min at room temperature and incubated overnight at 4 °C with primary antibodies. After incubating with horseradish peroxidase-conjugated secondary antibody (1:2000; Thermo, Waltham, MA), membranes were washed 3 times with PBST. Immunoreactive proteins were detected using ECL reagents and visualized by Syngene Bio Imaging (Synoptics, Cambridge, UK). Protein levels were quantified using Quantity One software (Bio-Rad).

### Apoptosis analysis by flow cytometry

Cell apoptosis was assessed using the FITC-Annexin V Apoptosis Detection Kit (Beyotime Institute of Biotechnology) and flow cytometry according to the manufacturer’s instructions. Cells were seeded into 6-well microplates and incubated overnight, and cells were treated with glucose and metformin for the indicated periods. Cells were collected and then incubated Annexin V-FITC and propidium iodide (PI) (Vybrant; Invitrogen, Karlsruhe, Germany) for 15 min at 37 °C in the dark. Apoptotic cells were quantitated by flow cytometry (Becton-Dickinson, Franklin Lakes, NJ, USA).

### Caspase 3 activity assay

Human ovarian cancer cells were treated with low glucose and metformin in different conditions. After harvesting cells, caspase 3 activity was measured using a colorimetric assay kit (Beyotime Biotechnology Limited Company, Shanghai, China) according to the manufacturer’s instructions.

### RNA interference

The knockdown of the ASK1 gene in SKOV3 cells was performed using siRNA. The ASK1 siRNA and negative control siRNA were obtained from GenePharma (Shanghai, China). The siRNA sequences were as follows: human ASK1 #1, 5’-GCCAACACUACAGUCAGGAAUUAAU-3′; human ASK1 #2, 5’-UGAAGCUAAGUAGUCUUCUUGGUAA-3′, and control siRNA (Scramble), 5’-ACGUGACACGUUCGGAGAAdTdT-3′ [32, 33]. Cell lines were transfected using Lipofectamine 2000 (Invitrogen).

### Mitochondrial membrane potential (MMP, ΔΨm) assay

Human ovarian cancer cells were treated with low glucose and metformin in different conditions. After harvesting, cells were exposed to JC-1 solution (Biotrend, Cologne, Germany) for 30 min at room temperature in the dark. Green fluorescence (520–530 nm) and red fluorescence (> 550 nm) were measured by flow cytometry (Becton-Dickinson, Franklin Lakes, NJ, USA).

### Mitochondrial fractionation

After treating with low glucose and metformin, human ovarian cancer cells were harvested, and the pure mitochondrial fraction was extracted using a Mitochondria Isolation Kit (Beyotime Biotechnology, Shanghai, China). The experiment was performed three times.

### Ovarian cancer tumor xenografts in female nude mice

Athymic BALB/c female nude mice (4 weeks old and weight of 20 g) were purchased from Vital River Laboratory Animal Technology Co. Ltd. (Beijing, China). A human ovarian cancer xenograft model in female nude mice was established through subcutaneously injecting SKOV3 cells into the right flank of each mouse. Mice were randomly divided into control (*n* = 5) and treatment groups (n = 5) as follows: control group; NQDI-1 alone (1 mg/kg/day by intraperitoneal injection); metformin alone (200–600 mg/kg/day by gavage); and combination of NQDI-1 and metformin. Treatment was started when the tumor reached approximately 150–200 mm^3^. Tumor diameter was measured every 3 days using a caliper, and the tumor volume was calculated according to the following formula: length × width × height × 0.5. After treatment with NQDI-1 or metformin, tumors were excised, and tumor weights were measured [34].

### Immunohistochemical staining

Immunohistochemical staining was performed on ovarian cancer tumor xenografts models [34]. The tissue sections of of xenograft tumour were deparaffinized with xylene and dehydration in a graded alcohol series. After, antigen retrieval by microwaving in pH 6.0 citrate buffer for 10 min, inhibiting endogenous peroxidase activity was performed. After blocking nonspecific binding, the slides were incubated with primary antibody overnight at 4 °C in a humidified container. According to the manufacturer’s recommendations, immunoreactivity was detected through using diaminobenzidine (DAB) staining. Brown signals mainly in the cytoplasm were defined positive reactions.

### Statistical analysis

All data represent at least 3 independent experiments and are presented as the means ± SD. Data were analyzed using one-way ANOVA. *P*-values less than 0.05 were considered statistically significant.

## Results

### Low glucose in culture medium enhances sensitivity of ovarian cancer cells to metformin

**Fig. 1:**
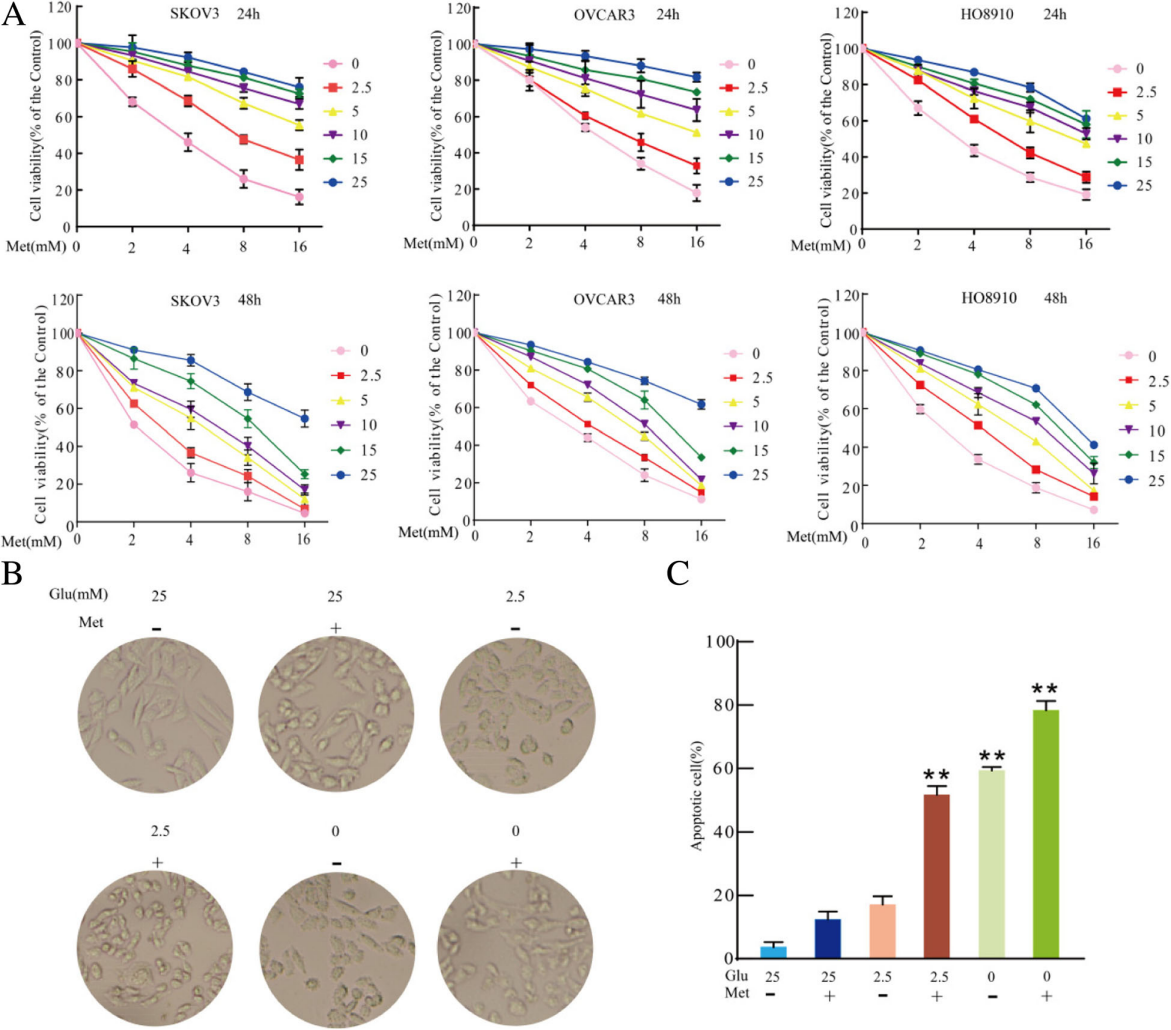
Low glucose and metformin in the culture medium inhibit the growth of human ovarian cancer cells. **a** Ovarian cancer cells, including SKOV3, OVCRA3 and HO8910, were treated with varying concentrations of glucose (abbreviated as Glu) and metformin (abbreviated as Met) for 24 h or 48 h. Cell viability was determined by MTT assay. Data are presented as mean ± SD (*n* = 3). **b** Cells were treated with varying glucose concentrations and 8 mM metformin for 24 h, and they were imaged using an optical microscope. **c** Percentage of apoptotic cells was detected by flow cytometry and quantitated. Data are presented as the mean ± SD (*n* = 3; **P < 0.01 versus control group)

Metformin has recently attracted attention as a potential cancer therapeutic and chemotherapy adjuvant [35]. Although metformin is effective as a cancer treatment, some cancer cells show resistance to metformin cytotoxicity [36, 37], which may be because the glucose concentration of the culture medium of cancer cells is higher than normal physiological conditions of 4–8 mM [9]. To investigate the effect of glucose and metformin, SKOV3, OVCAR3 and HO8910 ovarian cancer cells were treated with different concentrations of glucose combined with metformin, and the effect on cell survival was examined by MTT assays. As shown in Fig. 1a, decreasing glucose concentrations enhanced metformin cytotoxicity in SKOV3, OVCAR3 and HO8910 cells for 24 h or 48 h, especially for the combinations of 0 or 2.5 mM glucose and metformin. Under the condition of 25 mM glucose, the morphology of SKOV3 cells was normal for 24 h. With the combination of 25 mM glucose and metformin, SKOV3 cells showed slight shrinkage and became round for 24 h. Compared to 25 mM glucose with or without metformin, exposure to 2.5 or 0 mM glucose with or without metformin markedly increased the extent of cell shrinkage and roundness for 24 h (Fig. 1b). Based on the MTT assays and previous studies [9, 38], flow cytometry was used to examine apoptotic cell. Compared to 25 mM glucose alone or with metformin, exposure to 2.5 or 0 mM glucose with or without metformin induced cell apoptosis, especially in the combination treatments with 2.5 or 0 mM glucose and metformin (Fig. 1c). These results suggested that low glucose enhances metformin cytotoxicity in SKOV3 cells.

### Low glucose enhances metformin-induced apoptosis through a mitochondria-associated pathway

**Fig. 2:**
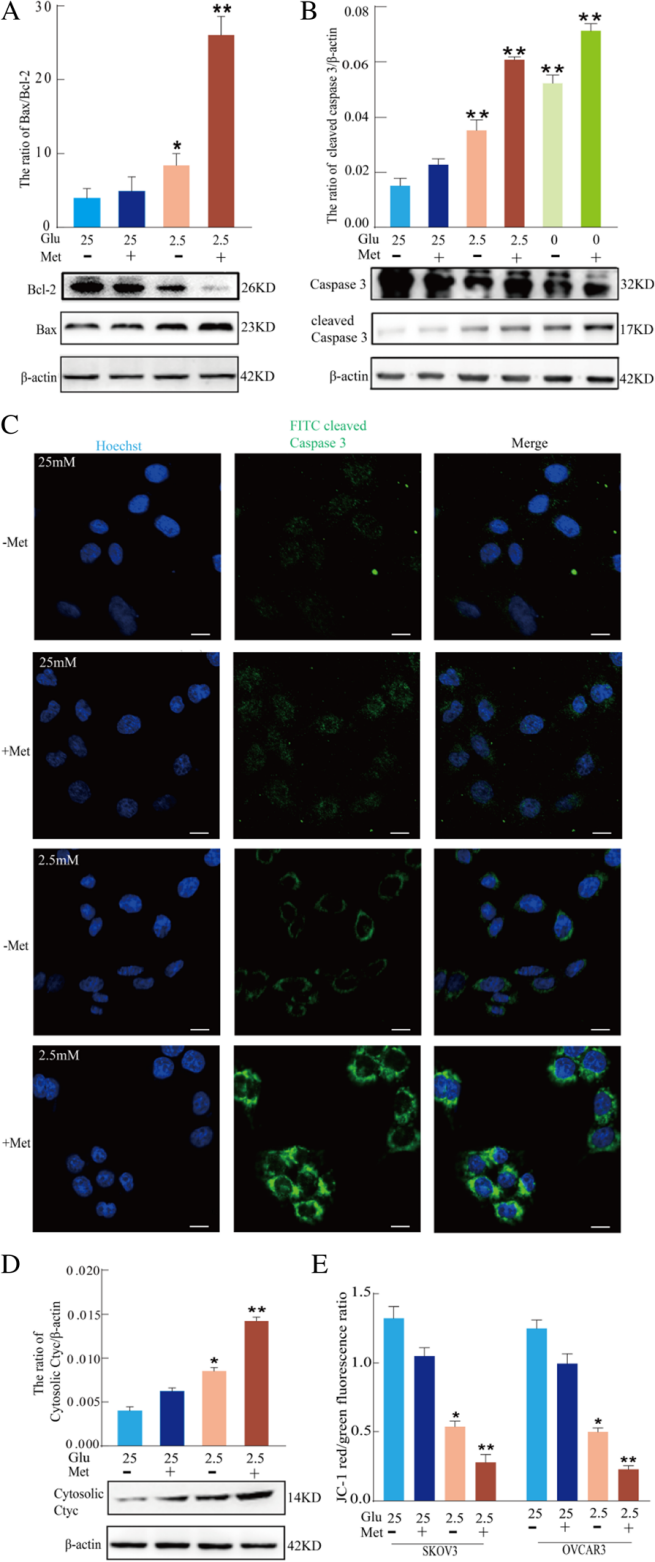
Low glucose enhances metformin-induced apoptosis through a mitochondria-associated pathway in human ovarian cancer cells. **a** Western blot assay of the expression of Bcl-2 and Bax proteins in SKOV3 cells treated with 25 mM or 2.5 mM glucose and 8 mM metformin for 24 h. Quantitation of the ratio of Bax/Bcl-2 protein. Data are presented as a mean ± SD (*n* = 3; *P < 0.05 and **P < 0.01). **b** Western blot assay of caspase-3 and cleaved caspase-3 protein expression in SKOV3 cells treated with varying concentrations of glucose and 8 mM metformin for 24 h. Quantitative analysis of cleaved caspase-3 protein expression. Data are presented as mean ± SD (*n* = 3; **P < 0.01 compared to the control group). **c** The expression of cleaved caspase 3 in SKOV3 cells was observed by confocal microscopy (bar, 10 μm). **d** Western blot assay of the expression of cytosolic cyt c protein in SKOV3 cells treated with 25 mM or 2.5 mM glucose and 8 mM metformin for 24 h. Quantitation of the level of cytosolic cyt c proteins. Data are presented as mean ± SD (*n* = 3; *P < 0.05 and **P < 0.01). **e** Flow cytometry analysis of JC-1 staining showed that 25 mM or 2.5 mM glucose and metformin in SKOV3 and OVCRA3 cells decreased the mitochondrial membrane potential after 24 h. The ratio of JC-1 red/green fluorescence was calculated using mean fluorescence intensity of each channel. Data are presented as mean ± SD (*n* = 3; *P < 0.05 and **P < 0.01)

It is generally considered that metformin acts mainly on mitochondria by decreasing cellular respiration through mild and specific inhibition of the respiratory-chain complex 1 (NADH: ubiquinone oxidoreductase) [36, 37]. To further investigate the mechanism of apoptosis induction by low glucose combined with metformin, the mitochondrial pathway was examined by western blot analysis of Bcl-2 and Bax expression. Compared to 25 mM glucose alone or with metformin, 2.5 mM glucose with metformin increased the ratio of Bax/Bcl-2 (Fig. 2a) in SKOV3 cells. Moreover, compared to 25 mM glucose with or without metformin, 2.5 mM glucose with metformin induced the expression of cleaved caspase-3 in SKOV3 cells as assessed using confocal microscopy (Fig. 2b and c). According to Fig. 2c 2.5 mM glucose with metformin enhanced the expression of cytosolic cytochrome c in SKOV3 cells (Fig. 2d). The level of mitochondrial membrane potential (Δ*ψ*m) was measured by flow cytometry following JC-1 staining. The combination of 2.5 mM glucose with metformin resulted in the loss of Δ*ψ*m in SKOV3 and OVCRA3 cells compared to 25 mM glucose with or without metformin (Fig. 2e). These findings suggested that mitochondrial dysregulation plays an important role in apoptosis induced by the combination of 2.5 mM glucose and metformin. These results also indicated that the combination of low glucose and metformin triggers cell apoptosis through the mitochondria-associated pathway.

### Low glucose and metformin-induced mitochondrial damage is associated with the ASK1/Noxa pathway

**Fig. 3:**
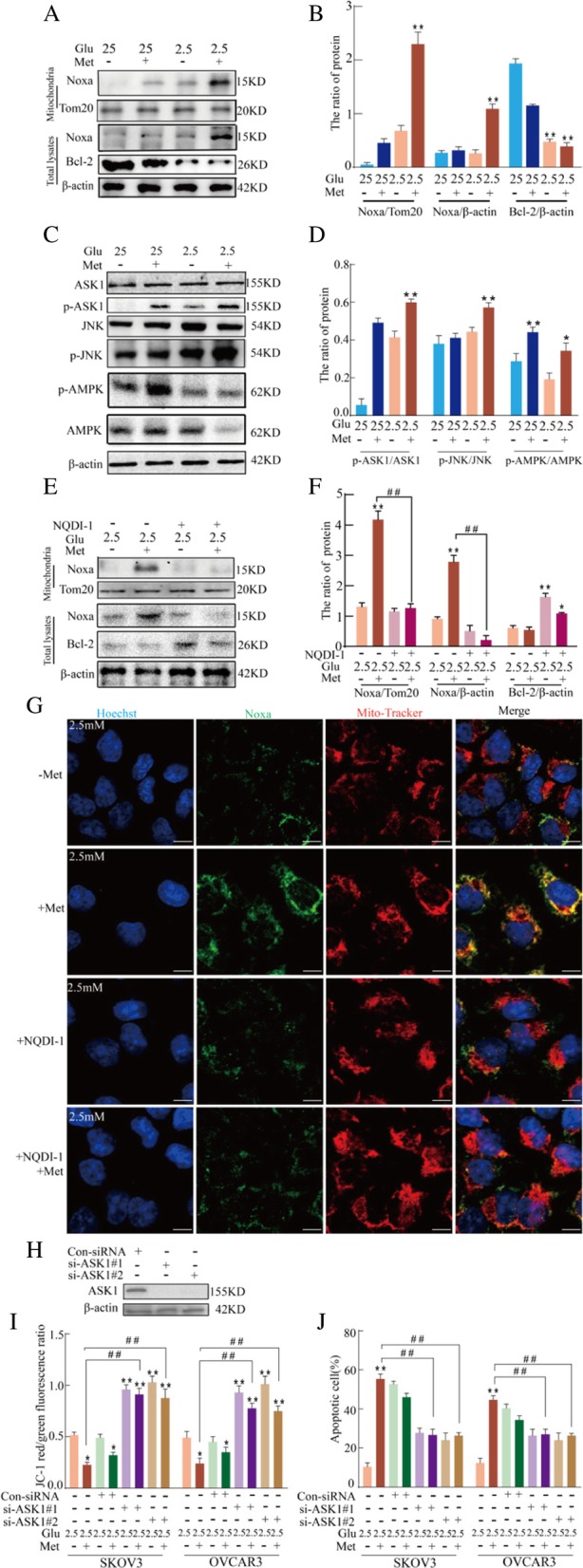
Low glucose and metformin-induced mitochondrial damage is associated with the ASK1/Noxa pathway. **a** Western blot assay of the expression of Noxa on the mitochondria, and total Noxa or Bcl-2 protein in SKOV3 cells treated with 25 mM or 2.5 mM glucose and 8 mM metformin for 24 h. **b** Quantitation of the ratio of Noxa/Tom20, Noxa/β-actin and Bcl-2/β-actin protein. Data are presented as mean ± SD (*n* = 3; **P < 0.01). **c** Western blot assay of the expression of ASK1, p-ASK1, JNK, and p-JNK proteins in SKOV3 cells treated with 25 mM or 2.5 mM glucose and metformin for 24 h. **d** Quantitation of the ratio of p-ASK1/ASK1 and p-JNK/JNK protein. Data are presented as mean ± SD (*n* = 3; **P < 0.01). (**e** and **f**) After pretreatment of SKOV3 cells with 10 μM NQDI-1, the expression of Noxa protein in the mitochondrial fraction and total Noxa or Bcl-2 protein was measured via western blot analysis (Mit. fraction) following treatment with low glucose and metformin for 24 h. The purity of the mitochondrial fraction was verified by the presence of the mitochondrial protein, Tom20. Quantitation of the ratio of Noxa and Bcl-2 protein. Data are presented as mean ± SD (*n* = 3; **P < 0.01). **g** Colocalization of Noxa and Mito-Tracker in SKOV3 cells as observed by confocal microscopy (bar, 10 μm). **h**-**j** ASK1 was knocked down in SKOV3 cells using small interfering RNA (siRNA). **h** Western blot analysis of ASK1 expression. **i** The effect of ASK1 on mitochondrial membrane potential in SKOV3 and OVCRA3 cells according to JC-1 staining and flow cytometric analysis. Data are presented as mean ± SD (n = 3; *P < 0.05 and **P < 0.01 versus 2.5 mM glucose alone; ^##^*P* < 0.01 versus the combination of 2.5 mM glucose and metformin). **j** The effect of ASK1 on cell apoptosis in SKOV3 and OVCRA3 cells as analyzed by flow cytometry. Data are presented as mean ± SD (*n* = 3; *P < 0.05 and **P < 0.01 versus 2.5 mM glucose alone; ^##^*P* < 0.01 versus combination of 2.5 mM glucose and metformin)

Noxa is a pro-apoptotic protein and is mainly associated with mitochondrial damage [38]. The present study investigated if low glucose and metformin-induced mitochondrial damage is associated with the expression and subcellular localization of Noxa protein. After treatment of SKOV3 and OVCRA3 cells with low glucose and metformin, the expression and subcellular localization of Noxa and Tom20 (mitochondrial marker) proteins were investigated by western blot analysis. Figure 3a and b shows that the total amount of the pro-apoptotic protein, Noxa, significantly increased after treating with the combination of 2.5 mM glucose and metformin in SKOV3 cells but that the expression of the anti-apoptotic protein, Bcl-2, was decreased. Consistently, immunoblot analysis also showed that Noxa protein was present in the mitochondrial fractions in SKOV3 cells treated with the combination of 2.5 mM glucose and metformin (Fig. 3a and b). To analyze key regulatory components of Noxa expression, apoptosis signaling regulating kinase 1 (ASK1)-dependent pathways were investigated because ASK1 activation is associated with cell apoptosis and is involved in mitochondrial damage [39, 40]. As shown in Fig. 3c and d, treatment of SKOV3 cells with 2.5 mM glucose and metformin induced the phosphorylation of ASK1 and JNK. Moreover, AMPK, a critical energy sensor of cellular energy homeostasis, was associated with metformin-induced glycolysis. Figure 3c and d shows that p-AMPK was significantly increased after treatment with the combination of 25 or 2.5 mM glucose and metformin in SKOV3 cells. In addition, the combination of 25 mM glucose and metformin had more obvious effects than the combination of 2.5 mM glucose and metformin. These data suggested that low glucose only inhibited partly activation of metformin-induced AMPK, but not as obvious as ASK1. To determine if ASK1 is involved in the subcellular localization of Noxa protein, the expression of ASK1 was suppressed by NQDI-1, a pharmacological inhibitor, in ovarian cells. Following pretreatment with NQDI-1, the total amount of Noxa was significantly decreased in SKOV3 cells treated with the combination of 2.5 mM glucose and metformin, and the expression of Bcl-2 was increased (Fig. 3e-f). After pretreatment with NQDI-1, the level of Noxa translocated to mitochondria was decreased in SKOV3 cells treated with the combination of 2.5 mM glucose and metformin using western blotting and confocal microscopy (Fig. 3e-g), suggesting that ASK1 plays an essential role in low glucose and metformin-induced subcellular localization of Noxa. To further determine the role of ASK1, the expression of ASK1 was suppressed using siRNA in SKOV3 cells. si-ASK1 reduced the expression of ASK1 protein compared to control cells (Fig. 3h). Moreover, siASK1 reduced the loss of Δψm and apoptotic ratio in SKOV3 and OVCRA3 cells treated with the combination of 2.5 mM glucose and metformin (Fig. 3i and j). These results suggested that low glucose and metformin-induced mitochondrial damage is the consequence of ASK1/Noxa pathway activation.

### Low glucose and metformin-induced activation of ASK1 is associated with ROS

**Fig. 4:**
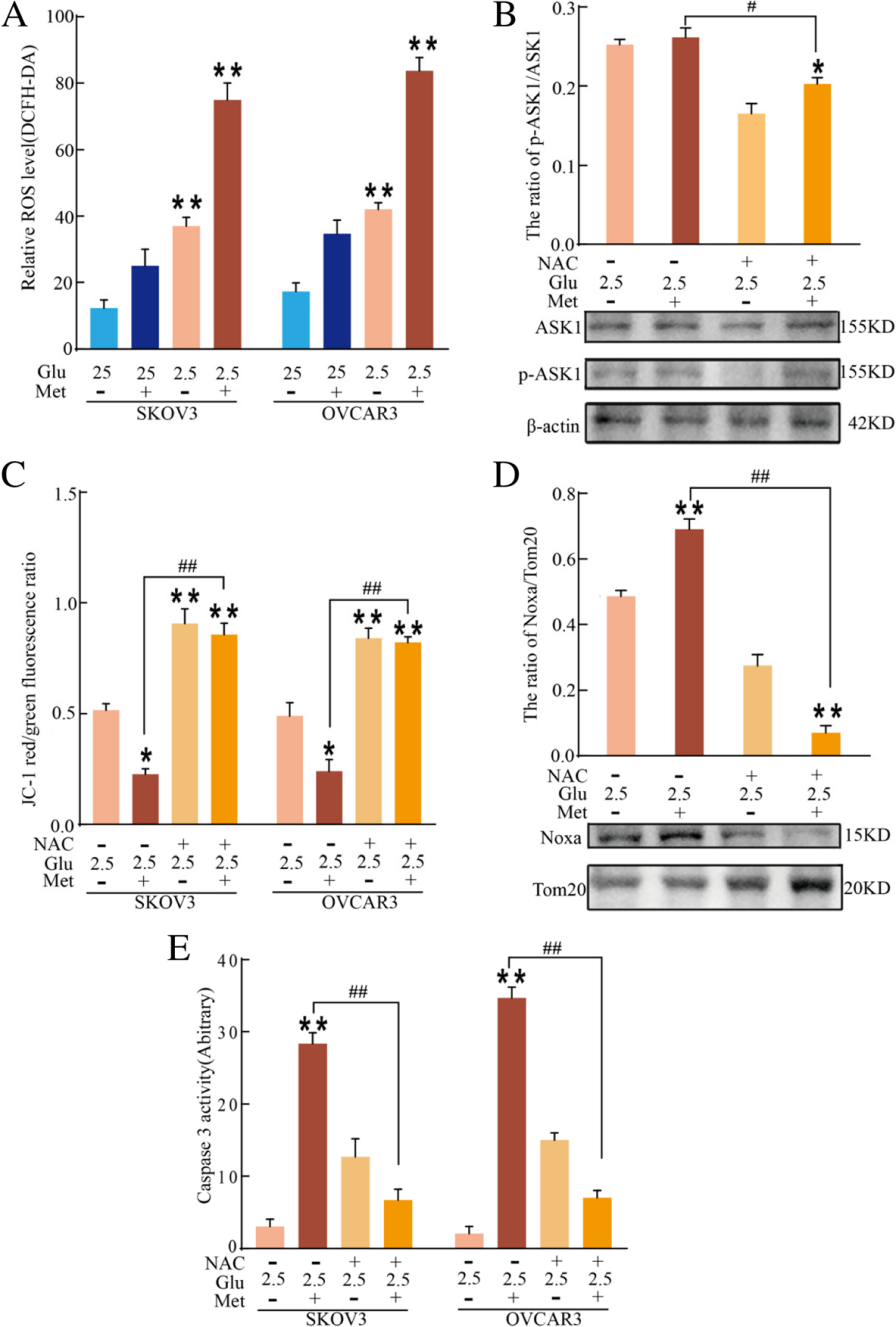
Low glucose and metformin-induced activation of ASK1 is associated with ROS. **a** Flow cytometric analysis of relative ROS level using DCFH-DA in SKOV3 and OVCRA3 cells treated with 25 mM or 2.5 mM glucose and metformin for 24 h. Data are presented as mean ± SD (*n* = 3; **P < 0.01). **b** Western blot analysis of the expression of ASK1 and p-ASK1 in SKOV3 cells following treatment with 25 mM or 2.5 mM glucose and metformin for 24 h. Quantitation of p-ASK1/ASK1 protein. Data are presented as mean ± SD (*n* = 3; *P < 0.05 versus 2.5 mM glucose alone; ^#^*P* < 0.05 versus combination of 2.5 mM glucose and metformin). **c** After SKOV3 and OVCRA3 cells were pretreated with 40 μM NAC, mitochondrial membrane potential was determined using JC-1 staining and flow cytometric analysis. Data are presented as mean ± SD (*n* = 3; *P < 0.05 and **P < 0.01 versus 2.5 mM glucose alone; ^##^*P* < 0.01 versus combination of 2.5 mM glucose and metformin). **d** After cells were pretreated with 40 μM NAC, the expression of Noxa protein in the mitochondrial fraction (Mit. fraction) of SKOV3 cells was analyzed by western blotting following treatment with 25 mM or 2.5 mM glucose and metformin for 24 h. Quantitation of Noxa protein in the mitochondrial fraction. Data are presented as mean ± SD (*n* = 3; **P < 0.01 versus 2.5 mM glucose alone; ^##^*P* < 0.01 versus combination of 2.5 mM glucose and metformin). **e** The caspase-3 activity in SKOV3 and OVCRA3 cells was measured by a colorimetric assay kit. Data are presented as mean ± SD (n = 3; **P < 0.01 versus 2.5 mM glucose alone; ^##^*P* < 0.01 versus combination of 2.5 mM glucose and metformin)

The activation of ASK1 is associated with accumulation of ROS and may further activate downstream signaling, including c-Jun-N-terminal kinase (JNK) [41]. The accumulation of ROS in SKOV3 and OVCRA3 cells was assessed using DCFH-CA and flow cytometry. According to Fig. 4a, ROS accumulation increased after treatment with the combination of 2.5 mM glucose and metformin. To analyze the role of ROS in the activation of ASK1 and JNK by the combination of 2.5 mM glucose and metformin, SKOV3 and OVCRA3 cells were pretreated with N-acetyl-l-cysteine (NAC). As shown Fig. 4b, western blotting demonstrated that the inhibition of ROS decreased the phosphorylation level of ASK1 in SKOV3 cells treated with the combination of 2.5 mM glucose and metformin. Thus, the accumulation of ROS may have been involved in the low glucose and metformin-induced loss of Δψm. Flow cytometric analyses showed that NAC markedly decreased the loss of Δ*ψ*m in SKOV3 and OVCRA3 cells treated with the combination of 2.5 mM glucose and metformin (Fig. 4c). Moreover, immunoblot analysis showed that NAC decreased the mitochondrial localization of Noxa induced by low glucose and metformin in SKOV3 cells (Fig. 4d). Moreover, NAC significantly inhibited caspase 3 activity in SKOV3 and OVCRA3 cells (Fig. 4e), suggesting that ROS accumulation is associated with Noxa-mediated mitochondrial damage. These results illustrated that ROS is involved in low glucose and metformin-induced activation of ASK1 and Noxa-mediated mitochondrial damage.

### Low glucose and metformin activate ER stress through ROS and ultimately trigger ER stress-associated apoptosis

**Fig. 5:**
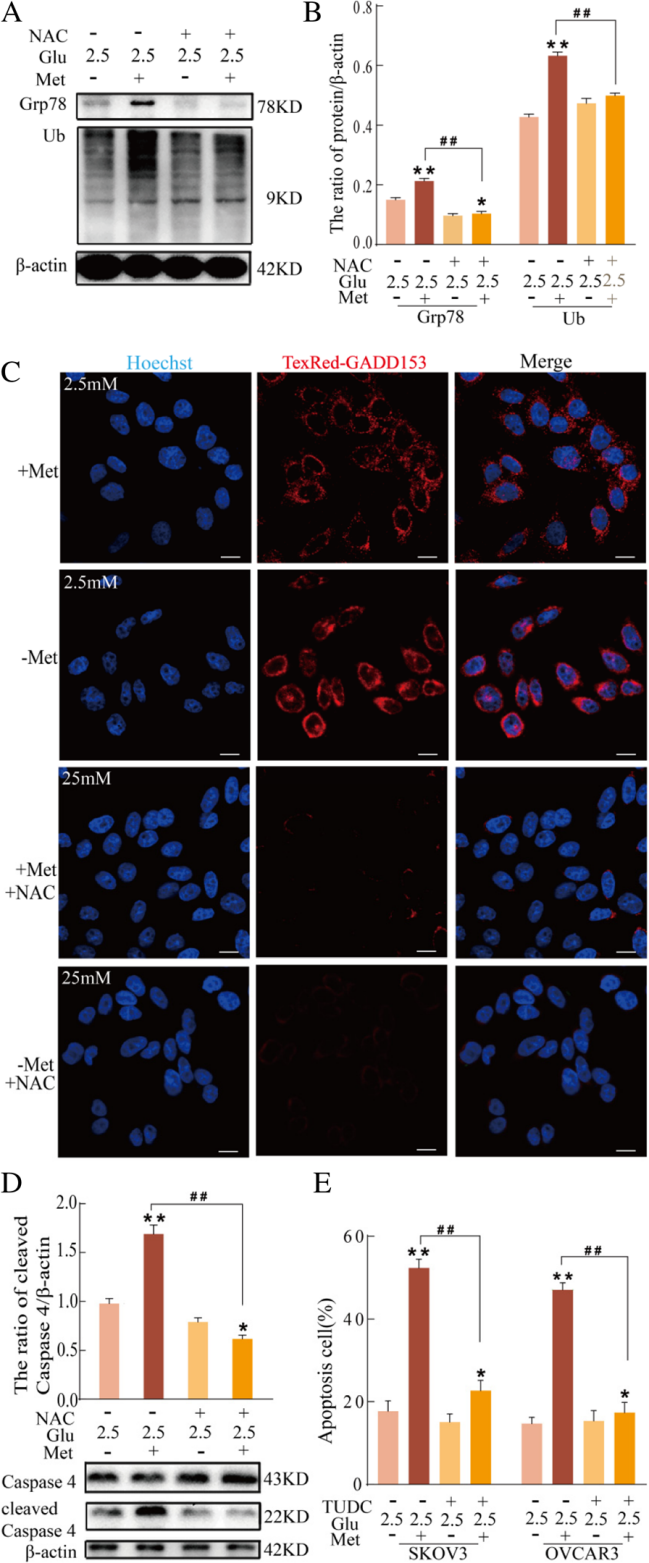
Low glucose and metformin activate ER stress and ultimately trigger ER stress-associated apoptosis. **a**-**d** SKOV3 cells were pretreated with 40 μM NAC. **a** Western blot analysis of the expression of Grp78 and Ub proteins in cells treated with 25 mM or 2.5 mM glucose and metformin for 24 h. **b** Quantitation of the ratio of Grp78 and Ub proteins. Data are presented as mean ± SD (n = 3; *P < 0.05 and **P < 0.01 versus 2.5 mM glucose alone; ^##^*P* < 0.01 versus combination of 2.5 mM glucose and metformin). **c** GADD153 expression was analyzed in SKOV3 cells treated with 25 mM or 2.5 mM glucose and metformin for 24 h (bar, 10 μm). **d** Western blot analysis of the expression of caspase 4 and cleaved caspase 4 proteins in SKOV3 cells treated with 25 mM or 2.5 mM glucose and metformin for 24 h. **e** After pretreatment of SKOV3 and OVCRA3 cells were with 500 μM TUDC, the ratio of apoptosis cells was determined by flow cytometry. Data are presented as the mean ± SD (n = 3; *P < 0.05 and **P < 0.01 versus 2.5 mM glucose alone; ^##^*P* < 0.01 versus combination of 2.5 mM glucose and metformin)

Previous studies have shown that metformin induces ER stress in cancer cells [42–44]. To further evaluate if low glucose and metformin–induced apoptosis is partially mediated by ER stress, Grp78, an ER-specific protein that indicates the occurrence of ER stress when upregulated, was examined. Compared to 25 mM glucose with or without metformin, 2.5 mM glucose and metformin further upregulated the levels of Grp78 and ubiquitinated proteins. In addition, NAC significantly decreased the expression of Grp78 and ubiquitinated proteins induced by 2.5 mM glucose and metformin (Fig. 5a and b). ER stress–induced cell death was analyzed by detecting GADD153 (CHOP). Confocal microscopy showed that the combination of 2.5 mM glucose with or without metformin enhanced GADD153 accumulation, especially the combination of 2.5 mM glucose and metformin. In addition, NAC significantly inhibited the expression of GADD153 induced by 2.5 mM glucose and metformin (Fig. 5c). In response to ER stress, caspase-4 is activated and processed. The activity of caspase-4 is reflected by its cleavage, which is required for ER stress-induced apoptosis (analogous to caspase-12 in murine cells). Cleaved caspase-4 was detected via immunoblot analysis. Compared to the combination of 25 mM glucose and metformin, the combination of 2.5 mM and metformin increased the expression of cleaved caspase 4 in SKOV3 cells, and NAC significantly inhibited the expression of cleaved caspase 4 induced by 2.5 mM glucose and metformin (Fig. 5d). These results suggested that low glucose and metformin induce ER stress and that activation of ER stress is associated with ROS. Moreover, flow cytometry showed that 500 M tauroursodeoxycholic acid sodium (TUDC; an inhibitor of ER stress) significantly decreased apoptosis induced by 2.5 mM glucose and metformin (Fig. 5e). These results suggested that low glucose may enhance metformin cytotoxicity through triggering ER stress-associated apoptosis and that ROS is involved in activation of ER stress.

### Low glucose and metformin activate ER stress through the ROS/ASK1/JNK pathway

**Fig. 6:**
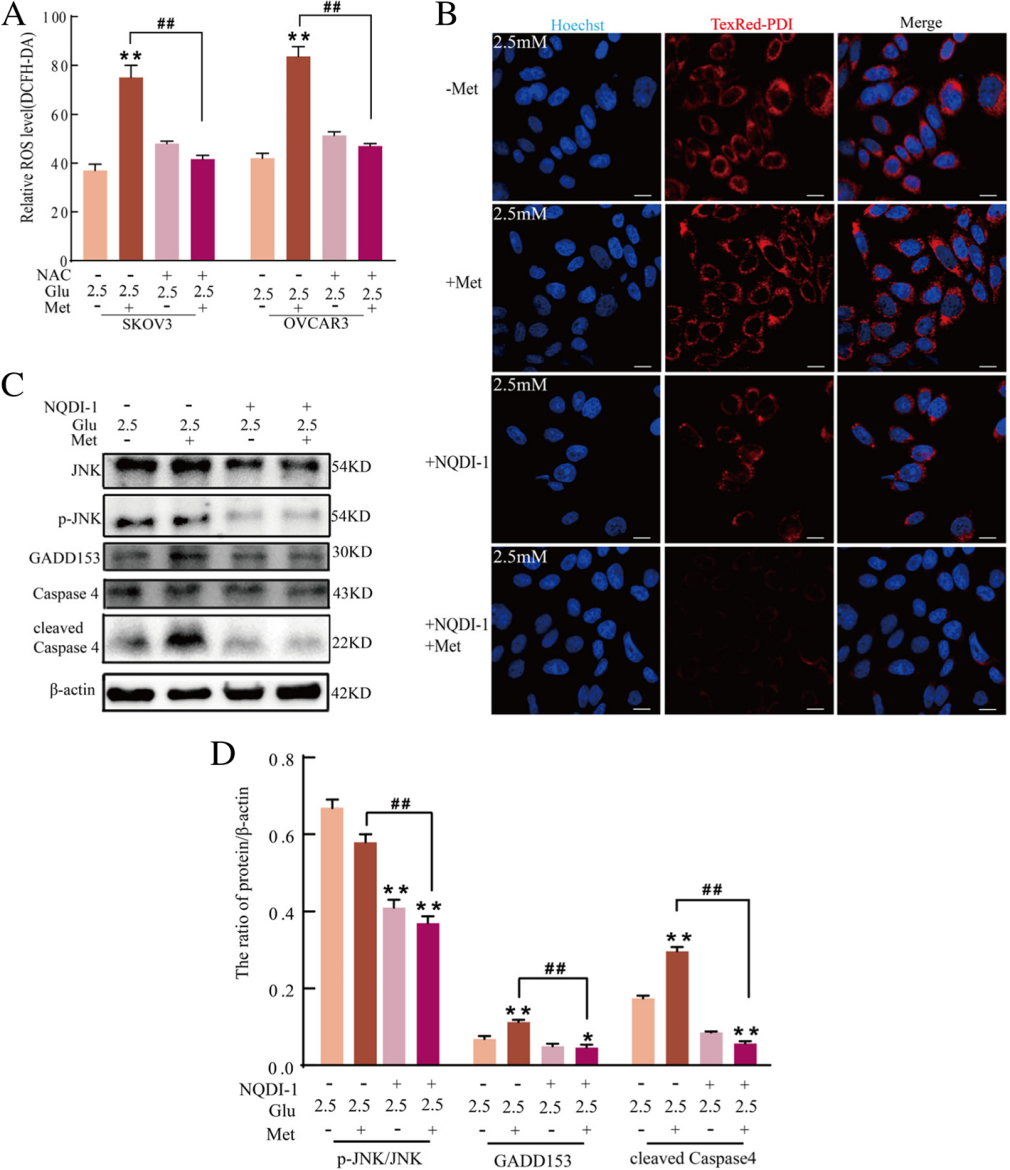
Low glucose and metformin activate ER stress through the ROS/ASK1/JNK pathway. **a** After SKOV3 and OVCRA3 cells were pretreated with 40 μM NAC, flow cytometry was used to analyze the relative ROS level using DCFH-DA in cells treated with 25 mM or 2.5 mM glucose and metformin for 24 h. Data are presented as a mean ± SD (n = 3; **P < 0.01 versus 2.5 mM glucose alone; ^##^*P* < 0.01 versus combination of 2.5 mM glucose and metformin). **b**-**d** After SKOV3 cells were pretreated with 10 μM NQDI-1, (**b**) the expression of PDI was observed in cells treated with 2.5 mM glucose and metformin for 24 h (bar, 10 μm). **c** Western blot analysis of the expression of GADD153, caspase 4, and cleaved caspase 4 in cells treated with 2.5 mM glucose and metformin for 24 h. **d** Quantitation of the ratio of GADD153 and cleaved Caspase 4 proteins. Data are presented as mean ± SD (n = 3; *P < 0.05 and **P < 0.01 versus 2.5 mM glucose alone; ^##^*P* < 0.01 versus combination of 2.5 mM glucose and metformin)

To further assess the role of ROS in low glucose and metformin-induced ER stress, SKOV3 and OVCAR3 cells were pretreated with NAC before exposure to low glucose and metformin. Several studies have reported that accumulation of ROS is involved in the modulation of the imbalance in ER homeostasis [45]. Therefore, SKOV3 and OVCAR3 cells were pretreated with NAC, and the level of ROS was detected. NAC significantly decreased the level of ROS in SKOV3 and OVCAR3 cells treated with the combination of 2.5 mM glucose and metformin (Fig. 6a). In addition, pretreatment of SKOV3 cells with NQDI-1 before exposure to low glucose and metformin markedly decreased the expression of PDI induced by the combination of 2.5 mM glucose and metformin (Fig. 6b). Moreover, western blotting demonstrated that NQDI-1 treatment of SKOV3 cells decreased the expression of p-JNK, GADD153 and cleaved caspase 4 induced by the combination of 2.5 mM glucose and metformin (Fig. 6c and d), suggesting that ASK1 is involved in low glucose and metformin-induced JNK activation and ER stress. These results suggested that low glucose and metformin induce cell apoptosis through triggering ER stress, which is associated with the ROS/ASK1/JNK pathway.

### ASK1 is involved in the anti-tumor effects of metformin: In vivo xenograft models

**Fig. 7:**
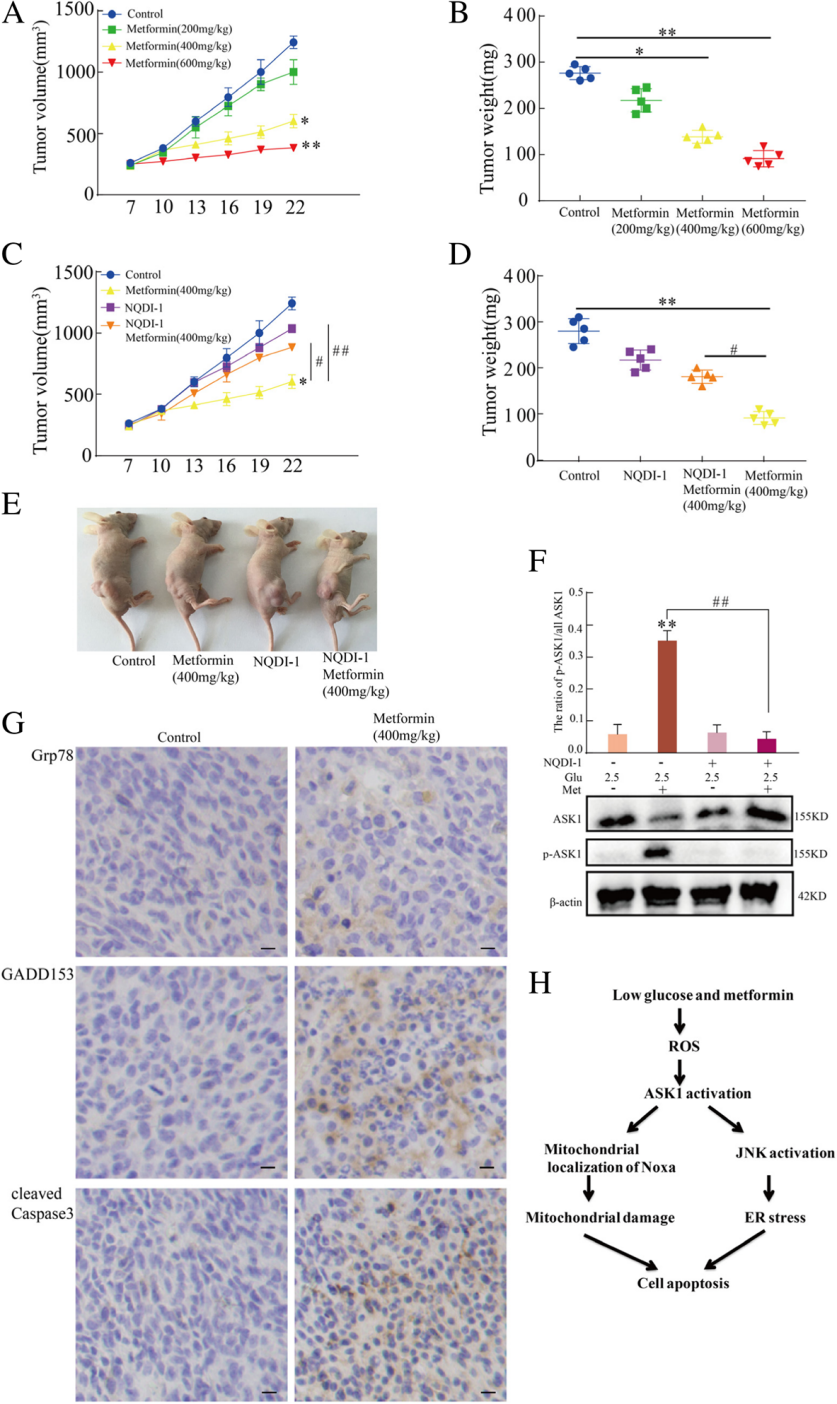
Metformin inhibits xenograft tumor growth through activation of ASK1. Tumors originating from SKOV3 cells reached 150–200 mm3 before treatment with NQDI-1, 200–600 mg/kg metformin, or a combination of NQDI-1 and metformin (*n* = 5 in each group). **a** The average tumor volume was diminished by different metformin doses (*P < 0.05 and **P < 0.01 versus control group). **b** The average tumor weights were measured (*P < 0.05 and **P < 0.01 versus control group). **c**-**e** NQDI-1 weakened the anti-tumor effect of metformin according to the change of tumor volume and weights (*P < 0.05 and **P < 0.01 versus control group; ^#^*P* < 0.05 and ^##^*P* < 0.01 versus metformin alone). **f** ASK1 and p-ASK1 levels according to western blot analysis of protein extracts from SKOV3 xenograft tumors (**P < 0.01 versus control group; ^##^*P* < 0.01 versus metformin alone). **g** The expression of Grp78, GADD153 and cleaved caspase 3 was detected through immunohistochemistry in SKOV3 xenograft tumors tissues. **h** Schematic diagram for low glucose and metformin-induced cell apoptosis

To evaluate the anti-tumor effect of metformin in ovarian cancer cells in vivo, nude mice with subcutaneous ovarian cancer xenografts were used as reported previously [46]. According to Fig. 7a and b, metformin decreased tumor volume and weight in a dose-dependent manner in vivo. The involvement of ASK1 in the anti-tumor effect of metformin in vivo was also investigated. As shown in Fig. 7c-e, the combination of NQDI-1 and metformin weakened the anti-tumor effect of metformin alone. Moreover, western blot analysis of tumors showed that metformin alone effectively induced ASK1 phosphorylation (Fig. 7f). Then, we detected the Grp78, GADD153 and cleaved caspase 3 through immunohistochemistry on ovarian cancer tumor xenografts models (Fig. 7g). We found metformin alone effectively increased the expression of Grp78, GADD153 and cleaved caspase 3, suggesting metformin in vivo in the xenograft model could induce ER stress and cell apoptosis. Collectively, these results showed that metformin induce ER stress and cell apoptosis, and ASK1 plays an important role in the anti-tumor effect of metformin in vivo.

## Discussion

Metformin is commonly used in the treatment of cancer and is thought to work through targeting mitochondria. However, recent studies have revealed that its cytotoxicity is enhanced by low glucose in various cancers, including ovarian cancer [9, 14, 47]. Glucose at a concentration of 25 mM, which is the normal level in cancer cell culture, can maintain typical plasma levels of 5–7 mM glucose and provide the optimal environment to promote cancer cell growth. As an anticancer agent, metformin weakly induces cancer cell apoptosis. However, under cell culture conditions with low glucose, the cytotoxicity of metformin increases cell apoptosis. The present study found that ovarian cancer cells were more sensitive to metformin at a glucose concentration of 2.5 mM compared to 25 mM. Glucose deprivation has been previously shown to increase metformin-induced cell death in breast cancer cells [27, 48, 49]. Low glucose and metformin-induced apoptosis of ovarian cancer cells was a consequence of ROS accumulation, which lead to mitochondria damage. ROS accumulation induced activation of ASK1, which, in turn, triggered the localization change in the pro-apoptotic protein, Noxa. Mitochondrial localization of Noxa protein lead to mitochondrial dysregulation, which was a key determinant for the initiation of apoptosis. Moreover, the present study demonstrated that ROS-ASK1 and JNK was associated with the modulation Noxa expression induced by low glucose and metformin. ASK1 activation was involved in the loss of ΔΨm, caspase 3 cleavage, and the subsequent release of cytochrome c. Therefore, the low glucose and metformin-induced mitochondrial associated apoptosis was a result of ROS-mediated ASK1 activation, which induced the phosphorylation of JNK, subsequently triggering the initiation of ER stress.

ER stress is identified as a critical factor in glucose deprivation–induced apoptosis through the ER stress pathway, and sustained and severe ER stress can trigger caspase-mediated apoptosis [27]. Oxidative injury, hypoglycemia, hypoxia, ER-Ca2^+^ depletion, high-fat diet, and viral infections may lead to an imbalance of ER homeostasis, thereby triggering ER stress. Moreover, the presence of ER stress induces mitochondrial dysfunction, which may activate cascades of the apoptotic signaling pathway [50–52]. However, the exact mechanisms of cell death induced by low glucose and metformin are unclear. According to the present data, metformin induced the elevation of ROS accumulation under the condition of 2.5 mM glucose, and NAC, a ROS scavenger, significantly reduced the degree of ER stress. Several studies have shown that a metformin-mediated ER stress response does not lead to cell apoptosis [42]. In contrast, other studies have reported that metformin triggers ER stress-dependent apoptosis [43]. The presence of prolonged or sustained ER stress activates the program of cell apoptosis [44]. Metformin reduces cellular respiration through inhibiting the respiratory-chain complex 1 (NADH: ubiquinone oxidoreductase) and, thus, affects mitochondrial function [14]. Moreover, recent studies have found that high glucose in the culture medium may partially cause metformin resistance and decrease the level of glucose-enhanced metformin cytotoxicity in cancer cells [48, 53]. In the present study, under the condition of 25 mM glucose, metformin-induced ER stress did not lead to cell apoptosis. However, low glucose enhanced metformin-mediated ER stress and cell apoptosis. TUDC, an inhibitor of ER stress, significantly decreased apoptosis induced by 2.5 mM glucose and metformin. Moreover, NQDI-1, an inhibitor of ASK1, reduced the ER stress induced by the combination of 2.5 mM glucose and metformin. Moreover, the activation of ASK1 lead to JNK phosphorylation. Consistently, metformin induced ER stress and cell apoptosis, and ASK1 played an important role in the anti-tumor effect of metformin on ovarian cancer tumor xenografts models, but we did not detect this effect in ovarian cancer cells elsewhere. Therefore, low glucose and metformin activated ER stress through the ROS/ASK1/JNK pathway in ovarian cancer cells.

In summary, ovarian cancer cells are insensitive to metformin under high glucose conditions, but low glucose enhances the cytotoxicity of metformin in these cells (Fig. 7g). Low glucose and metformin cause ROS accumulation, which, in turn, activates ASK1 and JNK. The activation of ASK1 triggers Noxa protein localization to mitochondria, further leading to mitochondrial dysregulation and ultimately cell apoptosis. Thus, low glucose and metformin induce mitochondrial dysregulation via ROS/ASK1/Noxa. ASK1-mediated activation of JNK induces sustained ER stress, and severe ER stress leads to apoptosis of ovarian cancer cells. Therefore, low glucose and metformin activate ER stress through the ROS/ASK1/JNK pathway. In conclusion, mitochondrial dysregulation and ER stress play an important role in low glucose–enhanced metformin cytotoxicity in human ovarian cancer cells. These results suggested that cell culture conditions may be an important determinant of metformin resistance in the treatment of some cancers.

## Conclusions

The present findings indicated that mitochondrial damage and ER stress play a crucial role in low glucose–enhanced metformin cytotoxicity in human ovarian cancer cells. Moreover, ASK1 is involved in low glucose and metformin-induced mitochondrial damage and ER stress. These results suggested that changes in cellular conditions combined with metformin treatment may represent a novel treatment strategy for the clinical treatment of cancer.

## Abbreviations

ASK1: Apoptosis signaling regulating kinase 1
DCFH-DA: Dichlorodihydrofluorescein diacetate
ER stress: Endoplasmic reticulum stress
Met: Metformin
MMP, ΔΨm: Mitochondrial membrane potential
MTT: (3-(4,5-dimethylthiazol-2-yl)-2,5-diphenyltetrazolium bromide
NAC: N-acetyl-l-cysteine
PBS: Phosphate-buffered saline
PI: Propidium iodide
ROS: Reactive oxygen species
si-ASK1: ASK1 siRNA
TUDC: Tauroursodeoxycholic acid
Ub: Ubiquitinated proteins
